# Compliance with wearing facemasks by university teaching staff during the second wave of COVID-19 pandemic: a cross sectional study

**DOI:** 10.1007/s44155-022-00011-3

**Published:** 2022-06-20

**Authors:** Mostafa Yosef, Fatma Amr Gamil Mokhtar, Wafaa Mohamed Hussein

**Affiliations:** Department of Community, Environmental, and Occupational Medicine, Faculty of Medicine, Ain Shams University, Cairo, Egypt

**Keywords:** COVID-19, Facemasks, Beliefs, Compliance, Barriers

## Abstract

**Objective:**

We aimed to explore compliance with and barriers to wearing facemasks at the workplace among university teaching staff in Egypt.

**Methods:**

An online survey was shared with teaching staff members at 11 public and 12 private Egyptian universities and high institutes, and 218 responses were received. All participants were asked about beliefs related to wearing facemasks. For participants who taught in-person classes, compliance with and barriers to wearing facemasks at the workplace were assessed. Compliance level was classified into: Non-compliance, inadequate and adequate, based on the degree of adherence to having facemasks on and not taking them off at five main work settings. We compared demographic characteristics, beliefs, and barriers scores across compliance levels.

**Results:**

Most participants (81.7%) believed that facemasks reduce infection risk to others and 74.3% believed facemasks can reduce risk to the wearer. Around 80% of the respondents who taught in-person classes wore facemasks, but only 37.8% met the criteria of adequate compliance. Difficulty breathing and impaired communication were cited as major barriers by 42.2% and 30.3% of in-person class tutors respectively. The risk of reporting COVID-19 like symptoms among non-compliant participants was double the risk among those with adequate compliance (45.9% vs 25.7% respectively). Adequate compliance was significantly associated with higher positive beliefs scores and lower barriers scores.

**Conclusion:**

Adequate compliance with wearing facemasks at the workplace was low. Addressing negative beliefs may improve compliance. Difficulty breathing, and impaired communication were important barriers, therefore we recommend replacing in-person interactions with online classes whenever applicable.

**Supplementary Information:**

The online version contains supplementary material available at 10.1007/s44155-022-00011-3.

## Background

Since it was first reported in December 2019, Coronavirus disease (COVID-19) caused by SARS-CoV-2 has resulted in 285 million confirmed cases globally including more than 5.5 million deaths by the end of December 2021 [1]. Egypt has reported its first COVID-19 case on the 14th of February 2020. By the end of December 2021, the country has officially recorded more than 384,000 confirmed cases with more than 21,000 deaths. Numbers of cases continue to rise in 2022 due to the rapidly spreading Omicron variant; fuelling a 5th wave of the pandemic [1, 2]. Like many low-middle income countries, Egypt has been struggling with under reporting and limited testing, therefore the actual numbers presumably exceed the officially reported numbers by a few folds [3–6].

Throughout the pandemic, several public health measures were recommended to control SARS-CoV-2 transmission including hand hygiene, social distancing, disinfecting surfaces and the use of facemasks [7–9]. More recently, several vaccines have been developed and approved for use in most countries [10, 11]. However, equitable distribution of vaccines, especially to developing countries poses a challenge [12]. Only 26.1% of the population in Egypt were fully vaccinated by the end of January 2022 [3]. Therefore, non-pharmaceutical public health measures remain a corner stone for the prevention and mitigation of the consecutive waves of the pandemic. Facemasks were found to be the most effective physical intervention interrupting community transmission of respiratory viruses such as coronaviruses, rhinoviruses, influenza viruses [13–16]. It was estimated that wearing facemasks in community settings may reduce primary infection risk with respiratory viruses by 6 to 15% [17]. Several modelling approaches have concluded that adherence to facemasks by 80% of the population or more could reduce the effective reproduction number below 1 leading to COVID-19 elimination, even if the facemasks were of suboptimal efficacy [18–22]. Both medical masks and masks made of cloth seem to reduce aerosol exposure, while offering a good measure of protection to the user [23–26]. Multiple studies have found the protection provided by surgical masks comparable to that provided by N95 respirators [27–29]. Even cloth masks were reported to be up to fivefold more effective than not wearing any [9, 24, 30].

The world health organization (WHO) recommends wearing facemasks in settings with poor ventilation or where physical distance of one metre is not possible, regardless of vaccination status or history of prior infection [31]. Several countries have instituted the mandatory use of facemasks and other face coverings in places where close contact is frequent and inevitable, particularly inside public transportation, shopping malls, workplaces, and educational facilities [27, 32].

Compliance with the use of facemask can vary between communities and individuals [23]. In a survey on 2097 participants in Iran, 55.7% stated that they always wear facemasks [33]. In a study where 9935 individuals were observed in the United States (US), compliance with wearing facemasks increased from 41.5% to over 90% after the enactment of mask mandates [23]. Yet, even when the majority wear facemasks in public, only a few wear them all the time as recommended [34]. Participants in a qualitative study in the US reported not wearing masks often around family and friends or co-workers [35]. These behaviours can fuel cluster infections in these groups which constitutes a common pattern for COVID-19 transmission [36, 37]. Women seem to be more likely than men to adopt protective behaviours like wearing facemasks during epidemics, including the current COVID-19 pandemic [23, 33, 38]. Older adults may have a higher tendency to comply with wearing facemasks due to the increased risk of COVID-19 complications [23, 39, 40].

Despite their evident benefit, wearing facemasks for long periods of time can pose some challenges such as: physical discomfort, complaints about the heat, glasses fogging, headache, perioral dermatitis and itchy rashes caused by the mask or its strings and difficulty breathing; especially with N95 respirators [41–45]. Social discomfort was also expressed, including aesthetic issues concerning not looking good in masks, in addition to confusion or misinformation and difficulty talking to other people [35, 44, 45]. Facemasks impair face recognition and communication, both verbal and non-verbal. They can block emotional signalling especially between teachers and learners in educational facilities [41, 46, 47].

In Egypt, there is little published research regarding the compliance with using facemasks. One survey on 726 participants showed that 99% believed that following public health measures can reduce SARS-CoV-2 transmission, but they did not measure actual practice [48]. In our study, we aimed to explore university teaching staff compliance with and barriers to wearing facemasks during in-person interactions at the workplace.

## Materials and methods

### Study design and study participants

We used a cross-sectional study design to measure the compliance of university teaching staff with wearing masks as well as barriers to wearing them. We recruited a convenience sample of teaching staff at 11 public and 12 private Egyptian universities and high institutes. Eligible participants were teaching staff at an Egyptian University or a higher institute with no exclusions based on age or gender. We invited participants to respond to a survey in Arabic language via posting a link to an online Microsoft form on several teaching staff social media groups. A sample size of 192 participants was calculated assuming a 50% prevalence for the main barrier to wearing facemasks, with a margin of error of 10% at 95% confidence level. A total of 218 teaching staff members responded to the survey.

### Study duration within the context of COVID-19 pandemic

Teaching staff were invited to respond to the survey throughout January 2021. The survey addressed staff use of facemasks during first semester of the academic year 2020–2021 that extended between the 17th of October and the 31st of December 2020. During that time, a blended learning modality was in place, mixing both online and in-person classes. By the 17th of October 2020, Egypt had reported 105,195 confirmed cases (and 6,099 deaths) since the beginning of the pandemic. The number of confirmed cases had risen to 136,644 (7576 deaths) on the 31st of December 2020 which marked the peak of the second wave in Egypt (with 1411 confirmed cases officially recorded on that day) [1]. Mandates for wearing facemasks in public settings (including markets, stores, public and private employees, public and private mass transportation, and banks) were imposed by the government since the 30th of May 2020 [49]. An immediate violation’s fine (50 EGP) was not imposed until the 3rd of January 2021 and it was enforced more strictly in public transportation rather than other public settings [50].

### Survey development and pilot

**Fig. 1:**
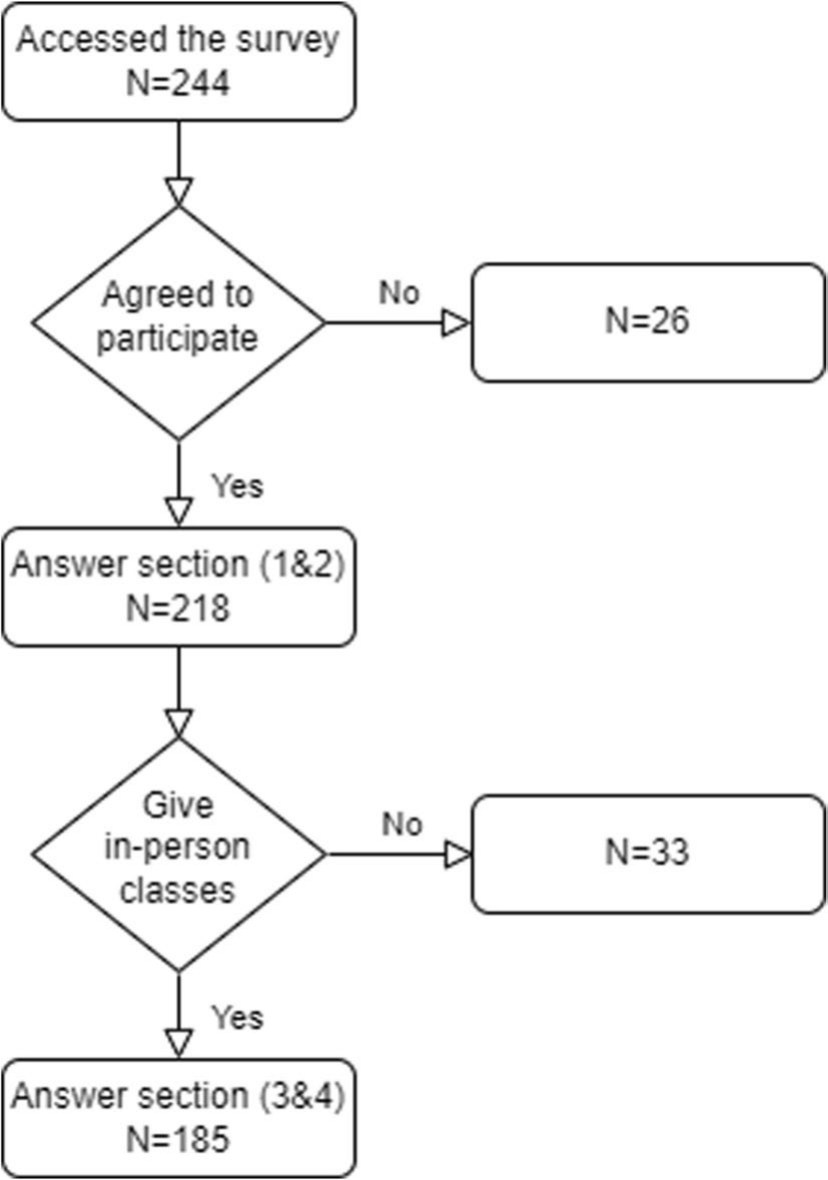
Flowchart of responses to the survey sections. Section (1): Demographic information and history of comorbidities, Section (2): Beliefs related to wearing facemasks, Section (3): Compliance with wearing facemasks, Section (4): Perceived barriers to wearing facemasks

Our survey consisted of four sections: (1) Demographic information and history of comorbidities, (2) Beliefs related to wearing facemasks, (3) Compliance with wearing facemasks in the workplace, and (4) Perceived barriers to wearing facemasks. Figure 1 shows the flow of responding to the four survey sections.

We searched the literature and identified eight items to examine participants’ beliefs in terms of: whether facemasks provided protection to the wearer and to others, whether there was a need to wear facemasks around students who wear/do not wear them, whether there was a need to wear facemasks in spacious rooms or around small number of students, whether all types of masks can reduce risk of infection, and if participants believed in superior protection by certain types of masks (e.g., N95 respirators) [23–25, 28, 30, 35]. All questions were graded on a five-degree Likert scale as follows: 1 = strongly disagree, 2 = disagree, 3 = neutral, 4 = agree and 5 = strongly agree. Beliefs’ score was calculated as the sum of the eight items in that section, with a maximum of 40 points. The scale for five items was reversed, so that an increasing beliefs score reflects an increasingly positive thinking.

To measure compliance with wearing facemasks, participants were asked to clarify on a 5-degree scale (1 = never, 2 = rarely, 3 = sometimes, 4 = often, 5 = always) “how often they wear a facemask” and “how often they tend to take it off” during in-person interactions in five main settings; namely: exam halls, lecture halls, small group classes, office hours and meetings with colleagues. A sixth option “Not applicable” was made available for participants who did not work in any given setting. Adequate compliance was defined as “always or often” wearing facemasks plus “never or rarely” taking them off in 75% or more of the settings where the participant works.

In the last section of the survey, we asked about eight of the common barriers to wearing facemasks derived from a study by Spitzer et al. [41]. The selected eight barriers were: difficulty breathing, fogged glasses, increased sense of heat or humidity, headache, skin irritation, impaired face recognition and identification, impaired communication, and impaired recognition of emotion signals. The magnitude of each barrier was measured on a four-degree Likert scale where 0 = not at all, 1 = minor barrier, 2 = moderate barrier and 3 = major barrier. Barriers’ score was calculated as the sum of the eight items in that section, with a maximum of 24 points.

### Survey translation and pilot

The survey was initially developed in English language. It was then translated into Arabic by the authors whose mother tongue is Arabic. The Arabic version was sent to an independent bilingual colleague (with an Arabic mother tongue) for back translation. The back translation was found comparable to the original English survey.

The Arabic version of the survey was piloted on 20 junior staff members to test the clarity and understandability of the survey wording. We measured the internal consistency of the beliefs and barriers scales. A Cronbach alpha statistic of 0.648 and 0.831 were obtained for beliefs scale and barriers scale respectively. Pilot data was not included in the study.

### Statistical analysis

Statistical analysis was performed using IBM SPSS Statistics for Windows, Version 25.0 (Armonk, NY: IBM Corp.). Mean and standard deviation were used to describe numerical variables, while frequency and percentage were used for to describe categorical variables. Chi-Square test was used to examine the relationship between compliance and sociodemographic characteristics. ANOVA test was used to compare mean scores across compliance levels among a subsample of 185 participants who taught face to face classes and Bonferroni Post Hoc test was used for pairwise comparisons. We compared the demographic characteristics of the total sample (N = 218) and the subsample (N = 185) and found no statistically significant difference [Supplementary table 1]. The level of statistical significance was considered as P ≤ 0.05.

## Results

We received 218 responses from university teaching staff members, of whom 171 (78.4%) were females. Most participants (156, 71.6%) worked at public universities and 153 (70.2%) were from medical specialties. The mean (± SD) age of the participants was 37.5 ± 9.3. The sample included 87 (39.9%) junior teaching assistants and 131 (60.1%) senior staff members of lecturers and professors (post PhD). Of all participants, 61 (28%) had at least one chronic disease and only 8 (3.7%) were smokers. When asked about history of COVID-19 infection, 67 (30.7%) of the participants reported suffering from COVID-19 like symptoms but only 15 (6.9%) had a confirmed COVID-19 diagnosis.

**Fig. 2:**
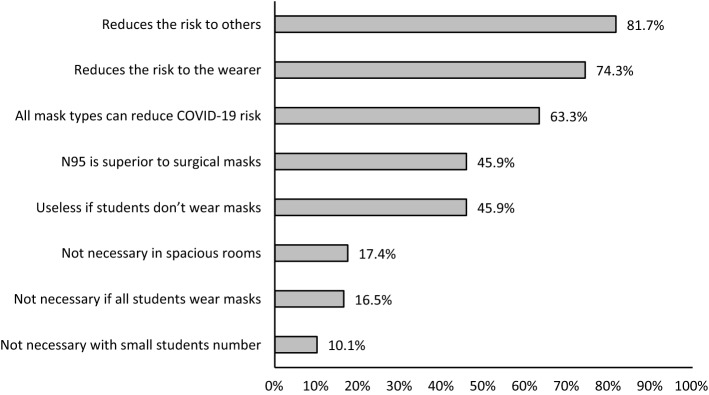
Proportion of participants who agree/strongly agree with statements about beliefs

All participants completed the section of beliefs regarding wearing facemasks during in-person interactions in the workplace. Figure 2 shows that the most participants (81.7%) agreed that facemasks reduce the risk to others and nearly 75% agreed facemasks can reduce risk to the wearer. Only a minority believed they did not need to wear facemasks in spacious rooms or when students wore them.

**Fig. 3:**
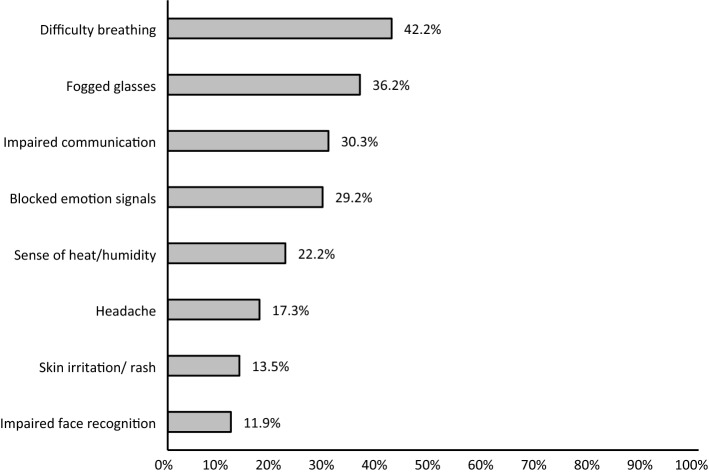
Major barriers to wearing facemasks reported by the participants (N = 185)

Most of the study participants (185, 84.9%) taught in-person classes, of whom the majority (148/185, 80%) wore facemasks during in-person interactions. However, only a little over one third (70/185, 37.8%) met the definition of adequate compliance and one fifth (37/185, 20%) did not wear facemasks at all and were labelled as non-complaint. The practice of the remaining participants (78/185, 42.2%) was labelled as inadequate compliance. Most of those who wore facemasks (126/148, 85.1%) used surgical masks either exclusively or in alternation with other types. Eleven participants (7.4%) wore only cloth masks, and an equal number wore only N95 respirators. Difficulty breathing, fogged glasses, impaired communication and blocked emotional signals were the commonest major barriers to wearing facemasks reported by the participants (Fig. 3).

We found no statistically significant association between adequate compliance and participants’ sociodemographic characteristics including sex, type of institution, specialty, experience, or having a chronic disease (Table 1).

**Table 1:** Adequate staff compliance with wearing facemasks in the workplace compared by their sociodemographic characteristics

		Total	Adequate compliance		OR (95%CI)
			Number	Row %	
Sex	Female^a^	144	53	36.8	
	Male	41	17	41.5	1.2 (0.6–2.5)
Specialty	Non-medical ^a^	54	17	31.5	
	Medical	131	53	40.5	1.5 (0.8–2.9)
Institution type	Private ^a^	53	19	35.8	
	Public	132	51	38.6	1.1 (0.6–2.2)
Experience	Senior ^a^	109	35	32.1	
	Junior	76	35	46.1	1.8 (0.9–3.3)
Chronic disease	No ^a^	135	50	37.0	
	Yes	50	20	40.0	1.1 (0.6–2.2)

Chi Square test was used

^a^Reference category

**Fig. 4:**
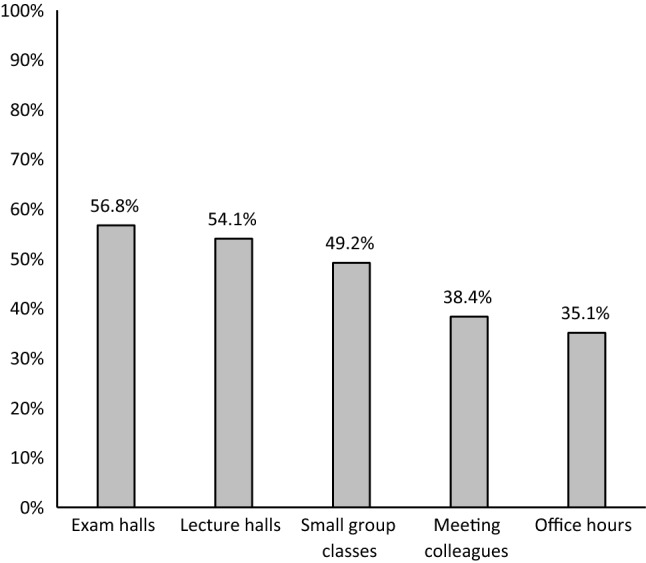
The proportion of “always/often wearing facemask and never/rarely taking them off” in each setting (N = 185)

When the five settings were compared (Fig. 4), we found that the proportion of adherence to “always/often wearing facemask and never/rarely taking them off” was highest in exam and lecture halls, and lowest at office hours. The difference was statically significant (Chi square = 13.531, p = 0.009).

**Fig. 5:**
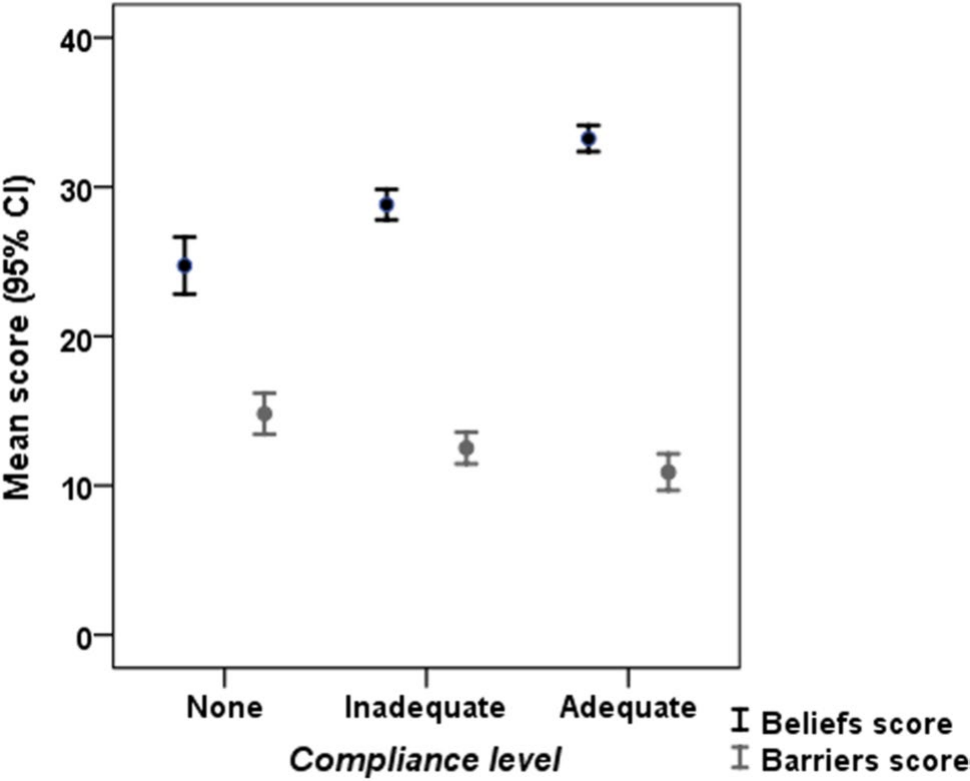
Mean (± 95%CI) for beliefs and barriers scores at each compliance level

We compared beliefs score across the three levels of compliance (Fig. 5) and the group with adequate compliance had the highest mean score (F = 45.848, p < 0.001). All pairwise comparisons between the three groups were statistically significant. Figure 5 shows that the mean Barriers’ score was highest among non-compliant participants compared to the other two groups (F = 8.242, p < 0.001). On pairwise comparisons, there was no statistically significant difference between adequate and inadequate compliance.

**Fig. 6:**
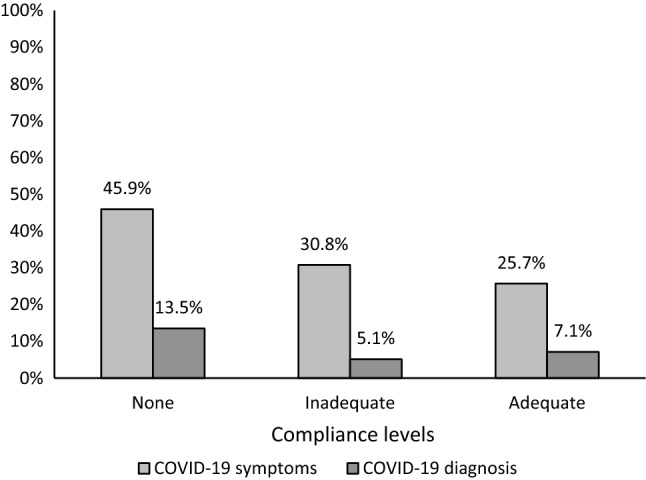
The proportion of participants who reported COVID-19 symptoms or diagnosis in each compliance level (N = 185)

Figure 6 shows that the risk of reporting COVID-19 like symptoms increased as the level of compliance decreased, being 45.9% among non-compliant participants compared to only 25.7% among those with adequate compliance (Chi square linear by linear association = 4.108, p = 0.043). Only 7.1% of those with adequate compliance had a confirmed COVID-19 diagnosis compared to 13.5% of the non-compliant group, but the difference was not statistically significant (Chi square linear by linear association = 0.877, p = 0.349).

## Discussion

Nearly 85% of the university teaching staff in our study gave in-person classes during the last three months of 2020. Mask mandates existed at that time, but they were not strictly enforced. While 80% of the in-person class tutors reported wearing facemasks, a little less than one third adhered to always/often wearing facemasks and never/rarely taking them off. Similarly, a survey in Iran reported that a little more than half of the participants “always” wore facemasks [33]. Similar surveys in the United States showed low prevalence of facemask use, especially in the absence of mask mandates, and that only a few wear them all the time [23, 34].

Previous studies have shown that women and older people were more likely to commit to wearing facemasks in public [23, 33, 38]. However, in the current study we found no statistically significant association between adequate compliance and certain demographic characteristics including age, sex or having a chronic disease.

Adherence to always/often wearing facemasks and never/rarely taking them off was generally low even in settings with typically large number of students as exam halls and lecture halls. Adherence declined further during meetings with colleagues and during office hours. A qualitative study in the United States has reported similar behaviour among employees at their work places [35]. This behaviour reflects staff’s low perception of risk colleagues which may result in cluster infections at the workplace [35, 37].

In agreement with previous research, participants in our study pointed out barriers to wearing facemasks, especially difficulty breathing and impaired communication, as students find it difficult to hear or understand what the lecturer says [41–45]. Impaired communication has been documented as a barrier to wearing facemasks in educational settings since facemasks block emotional signalling and impair verbal and nonverbal communication [41, 46].

In accordance with other studies, our respondents widely believed in the benefit of facemasks in protecting others around them [23–25]. Our respondents believed to a lesser extent in facemask ability to protect the wearer. Moreover, nearly half of them doubted the protection provided by wearing facemask if students in the room did not wear them as well. A qualitative study has highlighted uncertainty among the public about that benefit, thus stating the need to address it through clear educational messages [35]. Findings of the current study showed that participants who did not wear facemasks during in-person interactions had almost double the risk of reporting COVID-19 like symptoms compared to participants with adequate compliance. This goes in accordance with evidence from multiple reviews that supported the benefit of facemasks in protecting the wearer [24, 51]. The risk of getting a confirmed COVID-19 diagnosis in our study also seemed to be less  likely with adequate compliance, however we did not find the association statistically significant, possibly due to the small number of confirmed COVID-19 cases in our study.

Although several studies have concluded that wearing facemasks of any type can reduce COVID-19 transmission, only 60% of the participants in our study perceived that benefit [24, 25, 28–30, 51]. The participants in our study were equally divided regarding the belief that N95 respirators provided superior protection compared to surgical masks. This mirrors the controversy in the literature about the matter [9, 27–29]. A very small proportion of our respondents considered wearing facemasks unnecessary in spacious rooms or when all students wore masks or in the presence small numbers of students. Those participants had lower beliefs scores and were significantly less compliant with wearing facemasks. Although their numbers were small, these staff members are role models to students and to their younger colleagues, hence they could influence their beliefs and behaviours. More stress on awareness messages may be needed to address these risky perceptions, at least until other measures such as immunizing a sufficient proportion of the population takes place, which may allow for relaxing mask mandates in public settings in the future.

## Limitations

Due to the convenience sampling approach used in the study, we cannot exclude the risk of selection bias, especially the potential reluctance of non-compliant individuals to participate. Our assessment of beliefs, barriers and compliance with wearing facemask at the workplace was based on self-reporting with a potential risk of source bias or recall bias. Our cross-sectional study design did not allow for establishing the temporal association between adequate compliance and having COVID-19 like symptoms. COVID-19 symptoms and diagnosis were also self-reported. We did not formally assess the construct validity of the beliefs and barriers scales, although they seem to correlate well with the compliance levels. The internal consistency of the scales was acceptable, but test–retest reliability was not measured.

## Conclusions and recommendations

A little over one third of university staff members in our study showed adequate compliance with wearing facemask at the workplace. In-person interaction remains an integral part of the educational process in Egyptian universities, especially for teaching practical skills and for conducting examinations. Educational settings -especially in developing countries- host large numbers of students and teaching staff for long periods of time, with a significant risk of COVID-19 transmission. Hence, enhancing compliance with wearing facemasks is extremely vital to the safety of the teaching staff, the students, and the communities at large. Enforcing mask mandates in educational settings, along with continuous health education messages regarding the benefits of adequate compliance to wearing facemasks of any type are recommended. On the other hand, difficulty breathing, and impaired communication were important barriers to facemasks use. Reducing in-person interactions or replacing them with online classes may provide a temporary measure to counter the barriers to wearing facemask. In the long term, it is expected that the increasing roll out of COVID-19 vaccination might contribute to easing the mask mandates in the first place. This could provide a relief for non-compliant and partially compliant staff members struggling with barriers to wear facemasks.

## Supplementary Information

Below is the link to the electronic supplementary material.

Supplementary file1 (DOCX 17 KB)

## Data Availability

The dataset generated and analyzed during the current study is available from the corresponding author on reasonable request.

## Abbreviations

SARS-CoV-2: Severe acute respiratory syndrome coronavirus 2
COVID-19: Coronavirus disease 2019
N95: Respirators with 95% efficiency in removing 0.3 + micron non-oil-based particles
