# Supplementary material for: Compliance with wearing facemasks by university teaching staff during the second wave of COVID-19 pandemic: a cross sectional study
Source: Discov Soc Sci Health. 2022 Jun 20;2(1):8. doi: 10.1007/s44155-022-00011-3 (PMC9207867; doi:10.1007/s44155-022-00011-3)
Supplement: Supplementary file 1 — Supplementary file1 (DOCX 17 KB) [file 44155_2022_11_MOESM1_ESM.docx]

Table 1: Comparing demographic characteristics between the sample and the subsample

|  | | Sample (N=218) | | Subsample (N=185) | |  |
| --- | --- | --- | --- | --- | --- | --- |
|  |  | Mean | SD | Mean | SD | P value ^a^ |
| Age | | 37.5 | 9 | 37.3 | 9.1 | 0.782 |
| Beliefs-score | | 29.7 | 5.43 | 29.7 | 5.5 | 0.998 |
|  | | Number | % | Number | % | P value ^b^ |
| Sex | Male | 47 | 21.6 | 41 | 22.2 | 0.884 |
|  | Female | 171 | 78.4 | 144 | 77.8 |  |
| Specialty | Non-medical | 65 | 29.8 | 54 | 29.2 | 0.891 |
|  | Medical | 153 | 70.2 | 131 | 70.8 |  |
| Seniority | Junior | 87 | 39.9 | 76 | 41.1 | 0.811 |
|  | Senior | 131 | 60.1 | 109 | 58.9 |  |
| Institution type | Public | 156 | 71.6 | 132 | 71.4 | 0.963 |
|  | Private | 62 | 28.4 | 53 | 28.6 |  |
| Chronic disease | No | 157 | 72.0 | 135 | 73.0 | 0.831 |
|  | Yes | 61 | 28.0 | 50 | 27.0 |  |
| COVID-19 symptoms | No | 151 | 69.3 | 126 | 68.1 | 0.803 |
|  | Yes | 67 | 30.7 | 59 | 31.9 |  |
| COVID-19 diagnosis | No | 203 | 93.1 | 171 | 92.4 | 0.790 |
|  | Yes | 15 | 6.9 | 14 | 7.6 |  |

^a^ Independent samples t test was used

^b^ Ch square test was used

Significance level considered at p≤0.05
